# Multi-scale study of normal aging predicts novel late-onset Alzheimer's disease risk variants

**DOI:** 10.1186/1471-2105-16-S15-P11

**Published:** 2015-10-23

**Authors:** Sarah M Neuner, Lynda Wilmott, Matthew DeBoth, Thomas Shapaker, Jesse Ingels, Lu Lu, Rob Williams, Gerd Kempermann, Matthew Huentelman, Catherine C Kaczorowski

**Affiliations:** 1 Department of Anatomy and Neurobiology, University of Tennessee Health Science Center, Memphis, TN 38163, USA; 2 Translational Genomics Research Institute (TGEN), Phoenix, AZ 85004, USA; 3 Department of Genetics, Genomics, and Informatics, University of Tennessee Health Science Center, Memphis, TN 38163, USA; 4 Center for Regenerative Therapies, TechnischeUnivsersitat Dresden, Dresden, 01307, Germany

## Background

Alzheimer's disease (AD) is a neurodegenerative disorder characterized by severe memory impairment and accumulation of neuropathological amyloid plaques and tau tangles. By contrast, ‘normal’ age-associated cognitive decline is less severe and generally occurs in the absence of neuropathology. Many believe aging- and AD-related memory impairments result from separate etiologies, but this distinction is not entirely consistent with emerging evidence that both are linked to hippocampal dysfunction. As aging is the most significant risk factor for late-onset AD (LOAD), we hypothesize that memory deficits in both ‘normal’ aging and AD are driven in part by some common underlying mechanisms, which are exacerbated in AD by disease-specific insults such as neurodegeneration, neuroinflammation, and neuropathologies. In addition, heritability estimates suggest a strong genetic component (50-80%) in both conditions[1,2]. Thus, elucidating genetic correlates of memory decline in ‘normal’ aging may identify risk factors that influence susceptibility to LOAD.

## Materials and methods

Given that genetically diverse mouse models have emerged as a powerful way to study complex human traits, we conducted a multi-scalar analysis of two independent mouse models of aging to test this hypothesis[3]. We combined memory tests with proteomic, transcriptomic, and genomic data to generate a list of the top 30 candidates that correlate with memory impairments. To evaluate the translational potential of these candidates, we performed secondary analysis using published hippocampal transcript data from LOAD patients relative to age-matched non-demented controls[4].

## Results

Eighteen genes including *TRPC3, GABRA3, GABRA5, GABRB1, GABRB2, WDFY3,* and *GRM1* were significantly differentially expressed relative to disease status, suggesting they may play a role in the human disease process. In addition, we wanted to identify whether any of our candidate genes contained single nucleotide polymorphisms (SNPs) that could be used to predict an individual's susceptibility to LOAD. To test the ability of our multi-scalar approach to detect known LOAD risk genes, we searched our full dataset for published risk genes. We identified *APOE, SORL1, EPHA1, BIN1,* and *TREM2* as significantly differentially expressed relative to memory function in our aging models, validating our approach. We then tested our novel candidates against the freely available ADGC “TGEN2” dataset that is a clinical characterized and neuropathologically verified cohort[5,6]. Our analysis yielded four nominally significant novel putative risk variants (*GABRB1, GABRB2, GRM1*, and *WDFY3*) using data generated by our study of aging models.

## Conclusions

Future work will investigate functional significance of these SNPs and validate mechanistic relevance to cognitive decline during aging and AD. This work demonstrates the utility of studying ‘normal’ aging models to both better understand molecular mechanisms mediating memory function in diverse populations and to identify those candidates with the best potential to translate into effective treatments and predictive biomarkers for cognitive decline in elderly humans.
